# Comparative study on the effects of grain blending on functional compound content and in vitro biological activity

**DOI:** 10.1038/s41598-024-63660-1

**Published:** 2024-06-02

**Authors:** Narae Han, Koan Sik Woo, Jin Young Lee, Hyeon Gyu Lee, Junsoo Lee, Yu-Young Lee, Mihyang Kim, Moon Seok Kang, Hyun-Joo Kim

**Affiliations:** 1 Department of Central Area Crop Science, National Institute of Crop Science, Rural Development Administration, Suwon, 16613 Korea; 2 Bioenergy Crop Research Institute, National Institute of Crop Science, Rural Development Administration, Muan, 58545 Korea; 3 Department of Food and Nutrition, Hanyang University, Seoul, 04763 Korea; 4 Department of Food Science and Biotechnology, Chungbuk National University, Cheongju, 28644 Korea

**Keywords:** Anti-diabetic, Antioxidant, Polyphenols, Mixed grains, Amino acid, Organic acid, Biotechnology, Plant sciences

## Abstract

In this study, changes in bioactive compound contents and the in vitro biological activity of mixed grains, including oats, sorghum, finger millet, adzuki bean, and proso millet, with eight different blending ratios were investigated. The total phenolic compounds and flavonoid contents ranged from 14.43–16.53 mg gallic acid equivalent/g extract and 1.22–5.37 mg catechin equivalent/g extract, respectively, depending on the blending ratio. The DI-8 blend (30% oats, 30% sorghum, 15% finger millet, 15% adzuki bean, and 10% proso millet) exhibited relatively higher antioxidant and anti-diabetic effects than other blending samples. The levels of twelve amino acids and eight organic acids in the grain mixes were measured. Among the twenty metabolites, malonic acid, asparagine, oxalic acid, tartaric acid, and proline were identified as key metabolites across the blending samples. Moreover, the levels of lactic acid, oxalic acid, and malonic acid, which are positively correlated with α-glucosidase inhibition activity, were considerably higher in the DI-blending samples. The results of this study suggest that the DI-8 blend could be used as a functional ingredient as it has several bioactive compounds and biological activities, including anti-diabetic activity.

## Introduction

Recently, improving the quality of life and life expectancy of individuals has become an increasing concern, therefore there is a greater consumer demand for functional foods, which contain bioactive compounds that can enhance physiological functions as well as reduce disease risks^1^. Furthermore, consumers have shown increased interest in low-calorie foods due to concerns pertaining to obesity and chronic diseases. Therefore, there is a high demand for healthier, low-calorie foods in the food industry^2^.

Oats, sorghum, finger millet, adzuki bean, and proso millet are common grains in Korea and are considered as functional foods as they provide dietary fiber, proteins, minerals, and antioxidants that are required for human health^3–5^. Oats (*Avena sativa* L.) is mainly cultivated in USA and countries in Europe, and it is primarily used for animal feed due to its nutritional qualities^6^. Recently, oats consumption by humans has increased because of the high contents of phytochemicals, such as β-glucan, sterol, tocols, phytic acid, and avenanthramides, in oats. Oats can help to reduce the risk of metabolic syndromes, including cardiovascular disease, type 2 diabetes, and gastrointestinal disorders^7^. Sorghum (*Sorghum bicolor* L.) has been ranked as the world’s fifth most crucial grain crop and is predominantly used for food, feed, and biomass production^8^. The functional compounds in sorghum, such as phenolic acid, flavonoid, policosanols, phytosterols, stilbenes, and tannins, play an important role in human health by reducing risk possibility of inflammation, cancer, obesity, and chronic diseases^9^. Finger millet (*Eleusine coranana* L.) and proso millet (*Panicum miliaceum* L.) are widely grown species among different types of millet. Millet is the sixth most important grain worldwide and is a major source of protein and energy^10^. Although finger millet is considered an important grain as it is a staple food and is widely cultivated in Africa and South Asia, there is little research on finger millets^11^. Finger millet is a rich source of phytochemicals, particularly polyphenols (e.g., hydroxybenzoic acid, hydroxycinnamic acid, and flavonoids), which assist in preventing chronic disease^12,13^. A few decades ago, proso millet was recognized as an underutilized millet for food, however, the utilization of proso millet has increased due to their nutritional profiles (consisting of 60–70% starch, 10–17% protein, and 7% fiber), low glycemic index, and gluten-free property^14,15^. Furthermore, proso millet has great potential for protecting against of liver injury and cardiovascular disease. Additionally, proso millet is considered to be a beneficial food for health as it contains various antioxidant compounds including carotenoids, polyphenols, active polysaccharides, and α-tocophenol^16^. Adzuki bean (*Vigna angularis* var. nipponensis) is grown worldwide and is widely popular in East Asia^17^. Traditionally, adzuki beans are used as medicine as well as food because they contain bioactive compounds. Adzuki bean-derived polyphenols have anti-hypertensive, anti-obesity, antioxidant, and immunoregulatory effects^18,19^. Considering their health benefits, these grains may serve as attractive candidates for creating well-balanced food.

Due to increased interest in healthier foods and the nutritional excellence of grains, the consumption of cooked rice with various grains is gradually increasing in the Korean population, of which the diet was previously white rice-based^20^. Previous studies have focused on the effects of different compositions of cooked rice with mixed grains on physicochemical properties^21^, sensory effects^22^, and nutritional and functional value^23^. However, determining the composition and content of bioactive compounds in mixed grains is also crucial for understanding their biological activities.

Following previous research findings that revealed the health-promoting effect of phytochemicals, we considered that dietary intake of mixed grains containing phytochemicals will have a variety of beneficial effects on human health, especially anti-diabetic effects. In addition, we hypothesized that the blending ratios of mixed grains may influence their biological activities. Therefore, in this study, the bioactive compound contents (total polyphenol and flavonoid) and in vitro biological activity (antioxidant and anti-diabetic) of mixed grains with different blending ratios were compared, and the corresponding primary metabolite profile (amino acid and organic acid) was evaluated using gas chromatography-mass spectrometry (GC–MS). Oats (cv. Daeyang) and sorghum (cv. Sodamchal), which exhibited remarkable anti-diabetic effects in our previous studies^24,25^, were selected as the main ingredients, and finger millet (cv. Finger 1ho), adzuki bean (cv. Arari), and proso millet (cv. Geumsilchal) were added at different quantities based on the mixture design developed for the blending ratio. This approach could serve as a useful tool for elucidating the effects of blending ratios on the bioactive compound contents and biological activities of mixed grains.

## Material and methods

### Materials and reagents

Oats were obtained from an oats farmhouse (Jeongeup, Korea), and sorghum, finger millet, adzuki bean, and proso millet were cultivated in Suwon, Korea. These grain cultivars were selected due to being commonly grown cultivars in Korea. The compositions of the mixed grains are shown in Table 1. The mixed grains were ground using a blender and passed through a sieve (100 mesh) to obtain a fine powder with uniformly-sized particles.

**Table 1:** Blending ratios (%) of the mixed grains.

Code	Oats	Sorghum	Finger millet	Adzuki bean	Proso millet
DI–1	50	50	–	–	–
DI–2	40	40	20	–	–
DI–3	40	40	–	20	–
DI–4	40	40	10	10	–
DI–5	35	35	15	15	–
DI–6	30	30	20	20	–
DI–7	35	35	10	10	10
DI–8	30	30	15	15	10

All reagents and standard chemicals were purchased from Sigma-Aldrich (St. Louis, MO, USA). Nanopure water was obtained from a water purification system (Milli-Q Ad-vantage A10, Merck Millipore, Billerica, MA, USA).

### Bioactive compounds assay

The sample extraction and evaluation methods for analysis of total phenolic and flavonoid content were conducted according to the methods used in our previous study with slight modifications^26^. The ground sample (4 g) was mixed with 40 mL of 100% ethanol and was stirred for 24 h at room temperature. Next, the mixture was centrifuged (CR22N, Eppendorf Himac Technologies Co., Ltd., Ibaraki, Japan) at 10,000×*g* for 20 min, and the supernatant was evaporated in a rotary evaporator (SB-1200, EYELA Co., Ltd., Tokyo, Japan). The concentrated extract was re-dissolved in dimethyl sulfoxide (DMSO) (1:10, w/v) and used for the bioactive compounds assay. A modified Folin–Ciocalteu procedure and aluminum chloride method were used for the total phenolic and flavonoid contents assay. Briefly, 10 µL of extract was mixed with 200 µL of 2% Na_2_CO_3_ and 10 µL of 50% Folin–Ciocalteu reagent, and the mixture was incubated for 30 min at 25 ℃. Then, the absorbance was measured at 750 nm using an absorbance microplate reader (Elx 808, BioTek Inc., Winooski, VT, USA). For the total flavonoid assay, 75 µL of the extract was mixed with 300 µL of deionized water, 22.5 of µL 5% NaNO_2_, 45 µL of 10% AlCl_3_, and 150 µL of 1 M NaOH, and the mixture was incubated for 20 min at 25 ℃. Then, the absorbance was measured at 510 nm using an absorbance microplate reader.

### In vitro biological activity assay

The in vitro antioxidant free radical scavenging activity of the DI-blended was determined using 2,2′-azino-bis(3-ethylbenzothiazolin-6-sulfonic acid) (ABTS) according to the method described in our previous study with a slight modification^27^. Briefly, 10 µL of the extract was mixed with 200 µL of 7.4 mM ABTS solution, and the mixture was incubated for 60 min. Next, the absorbance was measured at 735 nm using an absorbance microplate reader. In addition, the reducing power of the DI-blended was measured using to the method described by Oyaizu^28^ with a slight modification. The extract (200 µL) was mixed with 500 µL of 50 mM phosphate buffer (pH 6.6) and 500 µL of 1% K_3_Fe(CN)_6_, and the mixture was incubated for 20 min at 50℃. Then, 500 µL of 10% trichloroacetic acid was added to the mixture. The mixture was centrifuged at 10,000×*g* for 10 min. After which, the supernatant (500 µL) was mixed with 500 µL of water and 100 µL of 0.1% FeCl_3_. After 5 min incubation, the absorbance was measured at 700 nm using an absorbance microplate reader.

To evaluate the mixture’s anti-diabetic effects, α-glucosidase inhibitory activity was analyzed following the enzymatic methods described by Tibbot and Skadsen^29^. The extract (10 µL) was mixed with 90 µL of 0.5 unit/mL α-glucosidase dissolved in 0.1 M sodium phosphate buffer (pH 7.0) and incubated for 20 min at 37 ℃. Next, 100 µL of 1.5 mM *p*-nitrophenyl-α-D-glucopyranoside was added to the mixture. After 6 min of incubation at 37 ℃ in the dark, the absorbance was measured at 405 nm using an absorbance microplate reader. Acarbose was used as the positive control.

### Analysis of the amino acids and organic acids in mixed grains using GC–MS

The amino and organic acids of 1 g of sample were extracted using a modified version of the method described by Han, et al.^30^ and Park, et al.^31^. One milliliter of methanol solution containing isoleucine and glycolic acid (to a final concentration of 0.2 mg/g), which were used as internal standard compounds, was added to the sample dissolved in 14 mL methanol, prior to heating at 70℃ for 25 min. Then, the samples were cooled to room temperature for 30 min, and 14 mL of deionized water and 7 mL of chloroform were added, followed by vortexing for 10 min. The samples were centrifuged at 3000×*g* for 20 min. The supernatant was then concentrated to a final volume of 0.1 mL using a rotary evaporator. An additional 1.5 mL methanol was added to recover the residual metabolites on the surface of the round-bottomed flask. The concentrates were dried in a vacuum oven (HyperVAC-2124, Hanil, Gyeonggido, Korea) at 35 ℃ for 5 h, then mixed with 150 μL of N,O-bis(trimethylsilyl)trifluoroacetamide containing 1% trimethylchlorosilane and 150 μL acetonitrile. Prior to GC–MS analysis, the samples were heated at 70 ℃ for 20 min, cooled for 10 min, and filtered through a 0.22-μm polyvinylidene fluoride syringe filter. GC–MS analysis was performed using a 7890B Gas Chromatograph system equipped with a 5977A mass selective detector (Agilent Technologies, Santa Clara, CA, USA). GC–MS analysis was conducted using the conditions described by Han et al.^30^.

### Statistical analysis

All values are presented as the mean and standard deviation determined from experiment replicates (n = 3), calculated using SigmaPlot 14.0 software (Systat Software, San Jose, CA, USA). Analysis of variance (ANOVA) was conducted using the general linear model procedure in SPSS statistical software version 18 (SPSS Inc., Chicago, IL, USA) to evaluate significant differences among the metabolites and biological activities in mixed grains due to the blending ratios. The samples were compared using Tukey’s multiple comparison test with a significance level at *p* < 0.05. Heatmap analysis was conducted using MetaboAnalyst 5.0 (https://www.metaboanalyst.ca/ accessed on 13 May 2023) of the normalized and log-transformed mean value of the relative content of the metabolites to clarify differences between the blending samples^32^.

## Results and discussion

### Comparison of bioactive compound contents and in vitro biological activity in mixed grains with different blending ratios

In this study, the highest total phenolic content was detected in DI-6 (16.53 mg), followed by that of DI-1 (15.84 mg) and DI-8 (15.65 mg) (Table 2). The total flavonoid contents in the mixed grains ranged from 1.22 mg (DI-1) to 5.37 mg (DI-8). DI-6 and DI-8 showed relatively higher contents of bioactive compounds compared to those of other treatments. To understand the functional compound content in DI-blended samples, we analyzed the functional compound content in each individual grain (Table 3). The results showed that sorghum had the highest total polyphenolic compound and flavonoid content, with 290.18 mg GAE/g extract and 110.55 mg CE/g extract, respectively. It is likely that the high total phenolic compound and flavonoid content in our DI-blended samples were mainly due to the high total phenolic compound and flavonoid content in the sorghum. In a previous study, the total phenolic compound and flavonoid content in oats (Daeyang), sorghum (Sodamchal), adzuki bean (Arari), and proso millet (Geumsilchal) were reported as 14.37, 235.21, 15.31, and 7.92 mg GAE/g residue, respectively, and these results are considered to be at a similar level to the findings of our study^33^.

**Table 2:** Bioactive compound contents in mixed grains with different blending ratios.

Code	Total phenolic content (mg GAE^1^/g extract)	Total flavonoid content (mg CE^2^/g extract)
DI–1	15.84 ± 0.21^b^	2.51 ± 0.49^bc^
DI–2	14.67 ± 0.00^ef^	1.22 ± 0.32^bc^
DI–3	14.53 ± 0.24^f^	2.55 ± 0.88^c^
DI–4	14.43 ± 0.08^f^	2.44 ± 0.74^bc^
DI–5	15.09 ± 0.24^de^	2.55 ± 0.78^bc^
DI–6	16.53 ± 0.08^a^	4.07 ± 0.81^ab^
DI–7	15.18 ± 0.21^cd^	3.26 ± 0.21^b^
DI–8	15.65 ± 0.14^bc^	5.37 ± 0.81^a^

Content is presented as the mean ± standard deviation of three replicates. Superscript letters in the same column indicate significant differences between blending samples at *p* < 0.05, determined using Tukey’s multiple comparison test.

^1^GAE, gallic acid equivalent; ^2^CE, catechin equivalent.

**Table 3:** Bioactive compound contents in each individual grains.

Grains	Total phenolic content (mg GAE^1^/g extract)	Total flavonoid content (mg CE^2^/g extract)
Oats	3.09 ± 0.40^c^	1.16 ± 0.15^c^
Sorghum	290.18 ± 17.95^a^	110.55 ± 5.41^a^
Finger millet	21.52 ± 0.31^bc^	14.76 ± 0.15^b^
Adzuki bean	28.74 ± 1.43^b^	16.16 ± 0.33^b^
Proso millet	3.04 ± 0.14^c^	0.15 ± 0.11^c^

Content is presented as the mean ± standard deviation of three replicates. Superscript letters in the same column indicate significant differences between grains at *p* < 0.05, determined using Tukey’s multiple comparison test.

^1^GAE, gallic acid equivalent; ^2^CE, catechin equivalent.

The results of in vitro antioxidant ability analysis are presented in Table 4. The antioxidant activities of the mixed grain extracts were remarkably affected by the blending ratio; the highest ABTS activity and reducing power were observed in DI-8 (72.05 mg TE/g) and DI-6 (0.507). Antioxidant activity is associated with antioxidant content, including phenolic compounds and flavonoids. Additionally, polyphenols, which are present in various grains, have beneficial biological effects, including antioxidant activity^27^. This result indicates that mixed grains, such as oats, sorghum, finger millet, adzuki bean, and proso millet, are rich sources of various polyphenols that possess an excellent antioxidant activity.

**Table 4:** In vitro antioxidant capacity and anti-diabetic effect of mixed grains with different blending ratios.

Code	ABTS activity (mg TE^1^/g)	Reducing power (Abs.^2^^)^ at 700 nm)	α-Glucosidase inhibition activity (%)
DI–1	67.52 ± 0.68^bc^	0.46 ± 0.01^c^	49.81 ± 0.09^b^
DI–2	67.25 ± 1.16^bc^	0.49 ± 0.01^b^	46.45 ± 1.53^c^
DI–3	69.33 ± 0.73^ab^	0.47 ± 0.00^c^	36.13 ± 1.16^e^
DI–4	63.81 ± 1.37^d^	0.46 ± 0.00^c^	41.61 ± 0.75^d^
DI–5	69.52 ± 0.99^ab^	0.50 ± 0.00^ab^	29.96 ± 1.16^f^
DI–6	65.74 ± 1.25^cd^	0.51 ± 0.01^a^	37.27 ± 0.62^e^
DI–7	66.57 ± 1.23^bcd^	0.46 ± 0.00^c^	45.86 ± 0.65^c^
DI–8	72.05 ± 1.05^a^	0.50 ± 0.00^ab^	54.48 ± 0.69^a^

Content is presented as the mean ± standard deviation of three replicates. Superscript letters in the same column indicate significant differences between blending samples at *p* < 0.05, determined using Tukey’s multiple comparison test.

^1^TF, Trolox equivalent; ^2^Abs., Absorbance.

The consumption of foods enriched with active ingredients may potentially reduce oxidative damage and prevent chronic diseases, including obesity, hypertension, and diabetes^3–5,34^. Thus, this study focused on the anti-diabetic effect of mixed grains, with different blending ratios; our results showed that mixed grains are a good source of antioxidants. α-Glucosidase is an enzyme associated with the hydrolysis of carbohydrates into glucose, and analysis of its inhibitory activity is widely used to evaluate the anti-diabetic effect of different products^35^. Accordingly, we found that the α-glucosidase inhibitory effect of mixed grains gradually declined as the percentage of oats and sorghum decreased; the inhibition rate of DI-1 (50% oats and 50% sorghum) was 49.81%, whereas that of DI-4 and DI-3, which contain 10% and 15% less oats and sorghum than DI-1, was 41.61% and 29.96%, respectively. However, the enzyme inhibition rate was significantly increased following the addition of proso millet (DI-7 and DI-8), despite the oats and sorghum ratios being diminished. DI-8 exhibited the highest α-glucosidase inhibitory effect (54.48%). Sorghum, proso millet, and adzuki bean extracts improve type 2 diabetes by increasing glucose absorption^33^. Mokashi et al.^36^ demonstrated that flavonoids enhance the glucose uptake rate of HepG2 cells via the insulin receptor substrate 1/Phosphoinositide 3-kinase/Protein kinase B pathway. In this study, the better anti-diabetic effect of DI-8 might be due to its higher flavonoid content compared to that of the other blends. The result of this study highlight the potentiaa of the DI-8 blend as a healthy food source too prevent various chronic diseases based on its excellent antioxidant and anti-diabetic activity.

### Amino acid, and organic acid profiling of mixed grains according to their blending ratios

We inbestigated the content of 12 amino acids and 8 organic acids in the mixed grains and discovered that the metabolite profile varied depending on the blending ratio. The relative values of the primary metabolites contents are presented in Table 5, and we observed significant differences (*p* < 0.05) in the mean contents across samples with different blending ratios.

**Table 5:** The relative levels (μg/g dry weight) of the metabolites identified in the mixed grains according to the blending ratios.

Metabolites	DI-1	DI-2	DI-3	DI-4	DI-5	DI-6	DI-7	DI-8
Amino acids								
Alanine	63.74^a^	51.91^ab^	58.43^ab^	53.04^ab^	52.61^ab^	47.05^b^	49.06^b^	50.46^b^
Valine	20.68^bc^	18.06^d^	23.17^a^	22.24^ab^	20.75^abc^	19.75^cd^	22.78^ab^	22.02^abc^
Leucine	6.69^ns^	5.12	6.17	5.49	6.67	5.41	4.56	5.78
Glycine	14.30^e^	16.61^de^	25.16^abc^	30.92^a^	20.93^cde^	20.72^cde^	28.14^ab^	22.66^bcd^
Serine	24.82^ns^	20.26	24.08	22.05	25.37	18.83	20.81	18.22
Threonine	14.91^c^	15.98^bc^	20.17^ab^	23.12^a^	19.64^abc^	15.21^bc^	19.24^abc^	16.29^bc^
β-Alanine	4.18^d^	5.80^d^	11.84^a^	8.53^c^	8.86^bc^	10.91^ab^	10.76^abc^	9.99^abc^
Proline	12.93^d^	16.53^cd^	32.14^bcd^	40.97^b^	22.28^bcd^	37.58^b^	68.54^a^	35.29^bc^
Aspartic acid	1.56^c^	13.52^b^	24.43^a^	25.44^a^	19.16^ab^	13.49^b^	3.24^c^	4.44^c^
γ-Aminobutyric acid	132.38^a^	95.23^b^	92.35^b^	79.37^bc^	75.00^bc^	54.32^c^	81.25^bc^	59.00^c^
Glutamic acid	20.71^b^	31.70^b^	17.90^b^	66.50^a^	20.19^b^	23.96^b^	38.91^ab^	40.57^ab^
Asparagine	10.93^cde^	38.84^a^	20.37^bcd^	30.98^ab^	25.38^abc^	18.91^bcd^	ND^e^	4.60^de^
Organic acids								
Lactic acid	5.17^b^	4.25^b^	6.70^ab^	5.45^b^	4.46^b^	4.98^b^	6.48^ab^	8.66^a^
Oxalic acid	ND^1^^c^	2.75^bc^	9.41^a^	4.04^abc^	3.62^abc^	2.75^bc^	7.72^ab^	8.61^ab^
Malonic acid	ND^d^	ND^d^	4.53^a^	1.80^c^	3.19^b^	4.63^a^	3.06^b^	4.88^a^
Succinic acid	23.83^ab^	20.45^ab^	25.21^a^	26.54^a^	22.65^ab^	19.80^ab^	14.04^b^	21.92^ab^
Glyceric acid	3.35^bc^	2.84^c^	4.16^abc^	4.52^ab^	3.78^bc^	3.54^bc^	5.14^a^	3.78^bc^
Fumaric acid	11.62^ns^	9.54	12.85	13.88	11.63	9.59	9.60	11.16
Malic acid	255.36^b^	254.40^b^	316.02^ab^	370.96^a^	294.00^ab^	243.13^bc^	142.95^c^	295.43^ab^
Tartaric acid	ND^d^	ND^d^	24.25^a^	7.91^c^	12.80^b^	17.16^b^	8.10^c^	15.09^b^

All the values are presented as the mean of three replicates. Superscript letters in the same row indicate a significant differences between blending samples at *p* < 0.05, determined using Tukey’s multiple comparison test. ns, means not significant.

^1^ND, not detected.

Free amino acids have an important role in the overall taste of food products, and their content and/or composition are involved in food quality and sensory attributes^37^. Additionally, some nutritionally nonessential amino acids, such as glutamic acid, glycine, arginine, and proline, play important roles in antioxidative responses as well as in the prevention and treatment of metabolic diseases, including obesity and diabetes^38^. Among the amino acids found in the mixed grains, γ-aminobutyric acid (GABA) was predominant in the samples regardless of the blending ratio. GABA is known for its health benefits, such as its ability to regulate cardiovascular function, lower blood pressure, and improve symptoms associated with various neurological disorders^39^. DI-1, a blending sample composed of 50% oats and 50% sorghum, showed the highest value of GABA content, and the value gradually declined in the samples as the percentage of oats and sorghum decreased. Meanwhile, the levels of β-alanine, which is structurally similar to GABA, were relatively higher in the blending samples containing 20% adzuki bean (DI-3 and DI-6) than that of the sample containing no adzuki bean (DI-1).

Organic acids, particularly tricarboxylic acid (TCA) cycle intermediates, such as succinic acid, fumaric acid, and malic acid, are crucial for various metabolic processes such as the biosynthesis of amino acids, fatty acids, and secondary metabolites^40^. The quantities of organic acids present in the mixed grains varied according to the blending ratio. Malic acid was the primary organic acid in all the blending samples, with DI-4 having the highest value (370.96 µg/g) and DI-7 having the lowest (142.95 µg/g) (*p* < 0.05). Succinic acid was the second most abundant organic acid in the mixed grains, with DI-4 having the highest value and DI-7 having the lowest. Oxalic acid was not detected in DI-1, but its concentration increased to 9.41 µg/g in DI-3. Moreover, the mixed grains that contained proso millet, namely DI-7 and DI-8, had a relatively higher oxalic acid content than that of the other blends, except for DI-3. Although malonic acid and tartaric acid were not detected in DI-1 and DI-2, their levels increased as the ratio of adzuki bean (DI-3 and DI-6) and proso millet (DI-8) increased. Glyceric acid is an essential intermediate in the glycolytic pathway^41^. However, its content was lower than that of the other organic acids in all the blending samples.

### Clustering and visualization of data

**Figure 1:**
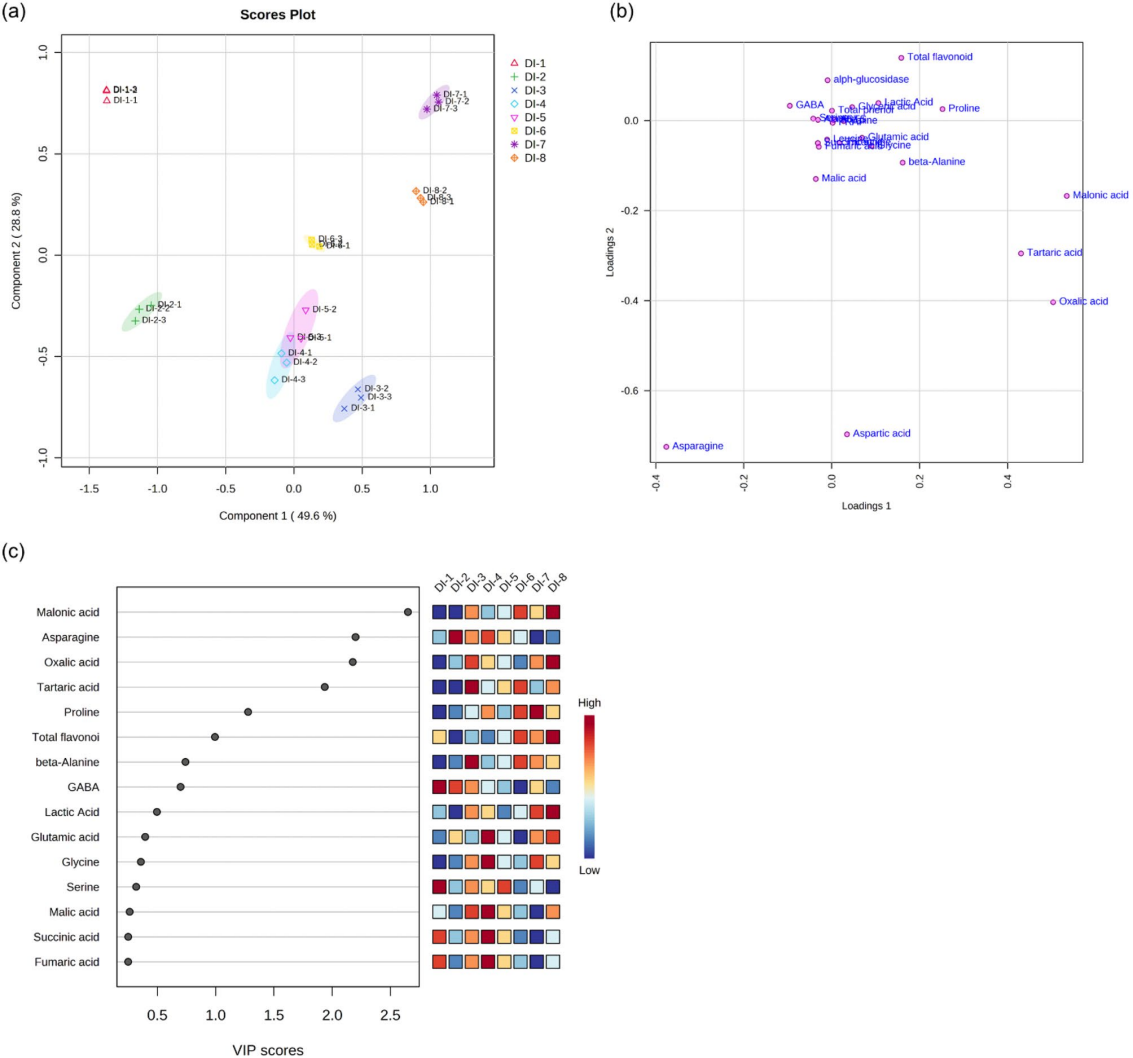
Partial least squared–discriminate analysis (PLS-DA) of different metabolites in mixed grains according to their blending ratio. (**a**) PLS-DA score plot; (**b**) PLS-DA loading plot; c, variable importance in projection (VIP) scores of the top 15 metabolites.

PLS-DA is a widely used technique in the field of chemometrics, which can be employed for predictive and descriptive modeling, and discriminative variable selection^42^. As depicted in Fig. 1a,b, the blending samples were clearly distinguished in the score plot obtained by combining component 1 (accounting for 49.6% of the total variance) and component 2 (accounting for 28.8% of the total variance). Variables important in projection (VIP) scores > 1 were identified as potential markers that contributed to group discrimination^43^. The key metabolites that differentiated the mixed blending samples based on blending ratio were malonic acid (2.6507), asparagine (2.2006), oxalic acid (2.1769), tartaric acid (1.9365), and proline (1.2787) (Fig. 1c).

**Figure 2:**
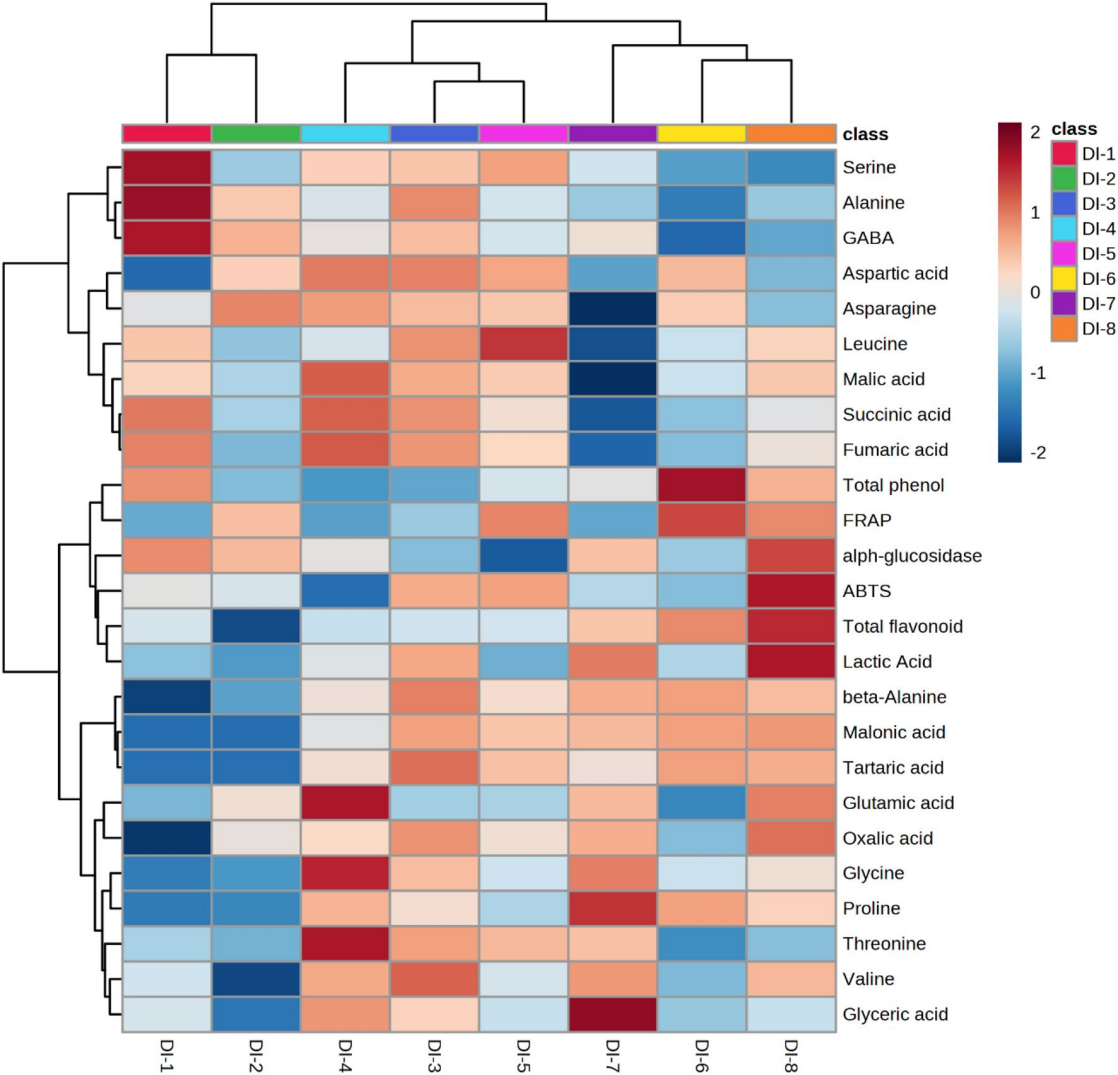
Hierarchical clustering and heatmap of metabolites and biological activity obtained from mixed grains according to their blending ratio. Red and blue indicate higher and lower values, respectively. The similarity measurement for clustering was based on the normalized data with Euclidean distance measurements and the Ward clustering algorithm.

The heatmap (Fig. 2) clearly visualizes the relationship between the mixed grains with different blending ratios, where high intensity values are shown in red. The clustering analysis grouped the blending samples into three distinct clusters: (1) DI-1 and DI-2, (2) DI-4, DI-3, and DI-5, and (3) DI-7, DI-6, and DI-8, which highlights the variation in their individual metabolite contents and biological activities. Cluster one (DI-1 and DI-2) exhibited lower amounts of lactic acid, β-alanine, malonic acid, tartaric acid, glutamic acid, oxalic acid, glycine, proline, threonine, valine, and glyceric acid compared to those in the other blending samples. Cluster two (DI-4, DI-3, and DI-5) had higher levels of aspartic acid, asparagine, malic acid, succinic acid, and fumaric acid compared to those of the other blending samples. Cluster three (DI-7, DI-6, and DI-8) showed higher amounts of β-alanine, malonic acid, and tartaric acid, and lower amounts of serine, alanine, GABA, succinic acid, and fumaric acid than those of the other blending ratios.

DI-8 exhibited the highest levels of bioactive compounds (total phenolic and flavonoid contents) and biological activity (antioxidant activity and α-glucosidase inhibitory effect) compared to those of the other blending samples, indicating its potential health benefits. In this study, the α-glucosidase inhibitory effects of the mixed grains were positively correlated (*p* < 0.05) with oxalic acid, lactic acid, total flavonoid, and glutamic acid contents (data not shown). Consequently, the higher anti-diabetic activity of DI-8 be due to those compounds. However, there is a dearth of studies exploring the individual flavonoid and polyphenol profiles of mixed grains containing oats, sorghum, finger millet, adzuki bean, and proso millet. Thus, additional research on secondary metabolite profiles in mixed grains to find out the major components involved in physiological activity are needed.

## Conclusions

In this study, we conducted a comprehensive analysis of the amino acid and organic acid composition of mixed grains using GC–MS and highlights the variation in biological activity and primary metabolite compositions in mixed grains depending on their blending ratio. In total, twenty metabolites were identified, with malonic acid, asparagine, oxalic acid, tartaric acid, and proline identified as the major components responsible for differences in the mixed grains with different blending ratios. Notably, the contents of malonic acid, tartaric acid, and oxalic acid were highest in DI-8 compared to those in the other blending samples, and DI-8 also demonstrated relatively higher antioxidant and anti-diabetic activities. Therefore, DI-8 may have potential as a functional food due to its rich content of bioactive compounds and superior biological activity. Overall, this study provides important insights into the composition and potential health benefits of mixed grains and may support the development of functional food products.

## Data Availability

The datasets used and/or analysed during the current study available from the corresponding author on reasonable request.

## Abbreviations

ABTS: 2,2′-Azino-bis(3-ethylbenzothiazoline-6-sulfonic acid)
ANOVA: Analysis of variance
CE: Catechin equivalent
DI: Diabetic inhibitor
DMSO: Dimethyl sulfoxide
GAE: Gallic acid equivalent
GC–MS: Gas chromatography–mass spectrometry
PLS-DA: Partial least squared-discriminate analysis
TCA: Tricarboxylic acid
TE: Trolox equivalent
TMS: Trimethylsilyl
VIP: Variables important in projection

## References

[1] Chen MF (2011). The joint moderating effect of health consciousness and healthy lifestyle on consumers' willingness to use functional foods in Taiwan. Appetite.

[2] Queiroz VAV (2018). A low calorie and nutritive sorghum powdered drink mix: Influence of tannin on the sensorial and functional properties. J. Cereal Sci..

[3] Ahola HG (2020). Immunochemical analysis of oat avenins in an oat cultivar and landrace collection. J. Cereal Sci..

[4] Girard AL, Awika JM (2018). Sorghum polyphenols and other bioactive components as functional and health promoting food ingredients. J. Cereal Sci..

[5] Kim H-J (2018). Quality and physicochemical characteristics of rice cooked along with various mixed grains and by following different cooking methods. Korean J. Food Nutr..

[6] Rasane P, Jha A, Sabikhi L, Kumar A, Unnikrishnan VS (2015). Nutritional advantages of oats and opportunities for its processing as value added foods—A review. J. Food Sci. Technol..

[7] Martínez-Villaluenga C, Peñas E (2017). Health benefits of oat: Current evidence and molecular mechanisms. Curr. Opin. Food Sci..

[8] Dahlberg, J. in *Sorghum: Methods and Protocols* (eds Zuo-Yu Zhao & Jeff Dahlberg) 269–277 (Springer, 2019).

[9] Khalid W (2022). Nutrients and bioactive compounds of *Sorghum bicolor* L. used to prepare functional foods: A review on the efficacy against different chronic disorders. Int. J. Food Prop..

[10] Chandra D (2016). Review of Finger millet (*Eleusine coracana* (L.) Gaertn): A power house of health benefiting nutrients. Food Sci. Hum. Wellness.

[11] Mbithi-Mwikya S, Van Camp J, Yiru Y, Huyghebaert A (2000). Nutrient and antinutrient changes in finger millet (*Eleusine coracan*) during sprouting. LWT Food Sci. Technol..

[12] Xiang J, Apea-Bah FB, Ndolo VU, Katundu MC, Beta T (2019). Profile of phenolic compounds and antioxidant activity of finger millet varieties. Food chem..

[13] Devi PB, Vijayabharathi R, Sathyabama S, Malleshi NG, Priyadarisini VB (2014). Health benefits of finger millet (*Eleusine coracana* L.) polyphenols and dietary fiber: A review. J. Food Sci. Technol..

[14] Singh M, Adedeji AA (2017). Characterization of hydrothermal and acid modified proso millet starch. LWT Food Sci. Technol..

[15] Bangar SP (2021). Proso-millet starch: Properties, functionality, and applications. Int. J. Biol. Macromol..

[16] Yuan Y (2022). Diversity of phenolics including hydroxycinnamic acid amide derivatives, phenolic acids contribute to antioxidant properties of proso millet. LWT.

[17] Wang Y (2022). Nutritional composition, efficacy, and processing of *Vigna angularis* (Adzuki bean) for the human diet: An overview. Molecules.

[18] Shi Z, Yao Y, Zhu Y, Ren G (2017). Nutritional composition and biological activities of 17 Chinese adzuki bean (*Vigna angularis*) varieties. Food Agr. Immunol..

[19] Kitano-Okada T (2012). Anti-obesity role of adzuki bean extract containing polyphenols: In vivo and in vitro effects. J. Sci. Food Agric..

[20] Han G, Lee Y (2014). Analysis of consumption status of cooked rice with different grains and related factorsin a Korean population: Based on data from 2011 Korean National Health and Nutritional Examination Survey (KNHANES). J. East Asian Soc. Diet. Life.

[21] Lee KH (2017). Physicochemical characteristics and antioxidant effects of cooked rice-added foxtail millet according to cooking method. J. Korean Soc. Food Sci. Nutr..

[22] Jung E-S (2010). Status of mixed grain diet by people with diabetes in Jeollabuk-do and sensory evaluation of different composition of mixed grains. J. Korean Soc. Food Sci. Nutr..

[23] Jang H-L (2013). Establishment of mixing ratio of multigrain rice for adolescent and aged people and its nutritional and functional estimation. J. Korean Soc. Food Sci. Nutr..

[24] Kim H-J (2020). Physicochemical properties, functional components, and physiological activities of sorghum cultivars. J. Korean Soc. Food Sci. Nutr..

[25] Song S, Lee YM, Lee YY, Yeum KJ (2021). Oat (*Avena sativa*) extract against oxidative stress-induced apoptosis in human keratinocytes. Molecules.

[26] Han N (2022). Effect of atmospheric-pressure plasma on functional compounds and physiological activities in peanut shells. Antioxidants.

[27] Han N (2022). Comparison of physicochemical characteristics, functional compounds, and physiological activities in adzuki bean cultivars. J. Korean Soc. Food Sci. Nutr..

[28] Oyaizu M (1986). Studies on products of browning reaction: Antioxidative activities of products of browning reaction prepared from glucosamine. Jpn. J. Nutr. Diet..

[29] Tibbot BK, Skadsen RW (1996). Molecular cloning and characterization of a gibberellin-inducible, putative α-glucosidase gene from barley. Plant Mol. Biol..

[30] Han N, Kim I, Kim J, Lee J (2021). Tissue-specific distribution of primary and secondary metabolites of Baemoochae (x*Brassicoraphanus*) and its changes as a function of developmental stages. Food Res. Int..

[31] Park MK (2010). Metabolite profiling of *Cheonggukjang*, a fermented soybean paste, during fermentation by gas chromatography-mass spectrometry and principal component analysis. Food Chem..

[32] Pang Z (2021). MetaboAnalyst 5.0: Narrowing the gap between raw spectra and functional insights. Nucl. Acids Res..

[33] Lee H (2020). Antioxidant and anti-diabetic activities of ethanol extracts of cereal grains and legumes. J. Korean Soc. Food Sci. Nutr..

[34] Zhou W (2023). Investigation of isoflavone constituents from tuber of Apios americana Medik and its protective effect against oxidative damage on RIN-m5F cells. Food Chem..

[35] Assefa ST (2019). Alpha glucosidase inhibitory activities of plants with focus on common vegetables. Plants.

[36] Mokashi P, Khanna A, Pandita N (2017). Flavonoids from Enicostema littorale blume enhances glucose uptake of cells in insulin resistant human liver cancer (HepG2) cell line via IRS-1/PI3K/Akt pathway. Biomed. Pharmacother..

[37] Zhang Z (2023). Ethylene treatment promotes umami taste-active amino acids accumulation of Torreya grandis nuts post-harvest by comparative chemical and transcript analyses. Food Chem..

[38] Wu G (2013). Functional amino acids in nutrition and health. Amino Acids.

[39] Sarasa SB (2020). A brief review on the non-protein amino Acid, gamma-amino butyric acid (GABA): Its production and role in microbes. Curr. Microbiol..

[40] Singh SK, Barnaby JY, Reddy VR, Sicher RC (2016). Varying response of the concentration and yield of soybean seed mineral elements, carbohydrates, organic acids, amino acids, protein, and oil to phosphorus starvation and CO_2_ enrichment. Front. Plant Sci..

[41] Habe H, Fukuoka T, Kitamoto D, Sakaki K (2009). Biotechnological production of D-glyceric acid and its application. Appl. Microbiol. Biotechnol..

[42] Lee LC, Liong C-Y, Jemain AA (2018). Partial least squares-discriminant analysis (PLS-DA) for classification of high-dimensional (HD) data: A review of contemporary practice strategies and knowledge gaps. Analyst.

[43] Choi R-Y, Ji M, Lee M-K, Paik M-J (2020). Metabolomics study of serum from a chronic alcohol-fed rat model following administration of defatted *Tenebrio molitor* larva fermentation extract. Metabolites.

